# Design of Stable α-Helical Peptides and Thermostable Proteins in Biotechnology and Biomedicine

**Published:** 2016

**Authors:** A.P. Yakimov, A.S. Afanaseva, M.A. Khodorkovskiy, M.G. Petukhov

**Affiliations:** Peter the Great St. Petersburg Polytechnic University, Polytechnicheskaya Str., 29, St. Petersburg 195251 , Russia; Petersburg Nuclear Physics Institute, National Research Center “Kurchatov Institute”, Orlova Roscha, 1, Gatchina, 188300, Russia

**Keywords:** α-helix, conformational stability, factors of thermal stability, membrane permeability, resistance to intracellular proteolysis

## Abstract

α-Helices are the most frequently occurring elements of the secondary structure in water-soluble globular proteins. Their increased conformational stability is among the main reasons for the high thermal stability of proteins in thermophilic bacteria. In addition, α-helices are often involved in protein interactions with other proteins, nucleic acids, and the lipids of cell membranes. That is why the highly stable α-helical peptides used as highly active and specific inhibitors of protein–protein and other interactions have recently found more applications in medicine. Several different approaches have been developed in recent years to improve the conformational stability of α-helical peptides and thermostable proteins, which will be discussed in this review. We also discuss the methods for improving the permeability of peptides and proteins across cellular membranes and their resistance to intracellular protease activity. Special attention is given to the SEQOPT method (http://mml.spbstu.ru/services/seqopt/), which is used to design conformationally stable short α-helices.

## INTRODUCTION

Not only have the numerous studies focused on α-helical peptides that have been conducted over the past quarter century contributed to a better understanding of protein folding into the native biologically active conformation, but also they are of significant interest for designing therapeutic agents that are efficient in treating diseases associated with a disruption of the protein–protein interaction [1, 2, 3]. Since the early 1990s, a large body of experimental data on the folding and stability of α-helices in monomeric peptides has been accumulated [4, 5]. These data demonstrate that the amino acid sequences of α-helices are usually not optimal for ensuring high conformational stability. This can be an important factor in preventing the accumulation of erroneous intermediate products in the folding of globular proteins. Hence, designing short α-helical peptides and proteins with sufficient conformational stability under specified ambient conditions (temperature, pH, and ionic strength) still remains an interesting problem of practical importance in protein engineering [1, 6].

The large amount of experimental data on the physical interactions that affect the stability of α-helices in proteins and monomeric peptides allows researchers to build theoretical models that describe α-helix–coil transitions and use them to elaborate new high-efficiency computational methods for designing α-helical peptides characterized by high conformational stability.

Stabilization of α-helices has been employed repeatedly to design industrially relevant enzymes that can work at elevated temperatures [7, 8]. This review discusses various methods for enhancing the thermal stability of globular proteins, including such molecular mechanisms as changing the amino acid composition of proteins in thermophilic organisms, inserting additional ion pairs and hydrogen bonds, using amino acids with an increased propensity to α-helical conformation, formation of additional disulfide bridges, strengthening of the hydrophobic interactions, introducing proline substitutions in loops, binding to metal ions, denser packing, etc.

## THE MOLECULAR MECHANISMS OF THERMAL STABILITY OF PROTEINS

Thermostable enzymes are used in many biotechnological processes, both in laboratory and in large-scale industrial production [9, 10].

Hyperthermophilic microorganisms that grow optimally at 80–110°C are the natural source of thermophilic proteins. These organisms, which were originally discovered in hot springs, mainly belong to the Archaea kingdom. The enzymes in these organisms also exhibit optimum activity at high temperatures ( > 70°C), and some of them are active at temperatures significantly higher than 100°C. Thermostable enzymes are usually inactive at temperatures below 40°C [11].

The ability to reliably predict the key physicochemical properties of mutant proteins, such as stability and solubility in water, is of paramount importance in molecular biology and biotechnology. A number of studies have been published where factors affecting the stability and solubility of proteins were investigated and models for predicting the consequences of point mutations in proteins were elaborated [11, 12, 13, 14]. Today, it is clear that there are many factors that can disrupt the stability or activity of a protein structure.

The features of the structural organization of thermostable proteins and enzymes that allow them to function at temperatures above 100°C have been intensively investigated both in experimental and fundamental studies, starting from the mid-1980s (according to the Medline database, a total of ~3,000 studies have been published) [12, 15, 16, 17, 18].

The discussion is based on a comparative analysis of homologous proteins from mesophilic and thermophilic microorganisms (further in this review referred to as mesophilic and thermophilic proteins, respectively). The thermostable proteins described by that time showed no specific features in their secondary or tertiary structures that were typical only of thermophiles as compared to their mesophilic analogues. However, the differences revealed at the level of their amino acid sequences were rather diverse. Such a variety of the characteristics of thermostable proteins can be grounded in the fact that thermophilicity is determined by a summation of the effects of various factors emerging due to a suitable combination of the weak interactions involved in protein stabilization.

It has been recently demonstrated that the mechanisms of adaptation to high temperatures in different organisms may depend on their evolutionary history [19]. Moreover, amino acid substitutions, presumably those associated with the thermal resistance of proteins, were found to often reside in α-helices [20, 21, 22]. Therefore, an analysis of the energy balance of molecular interactions inside α-helices may shed light on the reasons behind the stability of thermophilic proteins at high temperatures.

**Changes in the amino acid composition of proteins in thermophilic organisms**

Changes in the amino acid composition of proteins in thermophilic organisms compared to their mesophilic analogues were among the first factors affecting thermal stability that were studied [23, 24]. A statistical analysis demonstrated that glycine, serine, lysine, and the aspartic acid residues in thermophilic proteins are often replaced with alanine, threonine, arginine, and glutamic acid, respectively [25]. The function of these substitutions mainly consists in increasing the internal and reducing the external hydrophobicity of thermostable proteins. Moreover, these substitutions somewhat increase the stability of α-helices and the packing density of amino acids in thermostable proteins. A number of additional studies focused on various physical factors that can alter the amino acid composition of thermophilic proteins have recently been performed (see the review devoted to this topic [18]).

We have demonstrated in a series of studies [26, 27, 28] that the α-helices of thermostable proteins are in general more conformationally stable than the identical α-helices of highly homologous proteins in mesophilic and psychrophilic bacteria. The composition of the α-helices of thermostable proteins changes due to an increase in their content of amino acids with a high propensity to form α-helices (alanine and leucine) and, therefore, a decrease in their content of β-branched residues (valine, isoleucine, and threonine). Furthermore, a significant rise in the abundance of amino acids that can stabilize α-helices through strong interactions between their side chains and the side chains of other amino acids (e.g., glutamic acid and arginine) was detected. Similar findings were also made in a study performed by a different research group; particularly, a significant decrease in the content of β-branched residues with a low tendency to form α-helices in thermophilic proteins was also revealed [29].

Matthews et al. [30] demonstrated that the introduction of proline residues increases the thermal stability of a protein due to a decrease in the entropy of the unfolded state.

Additional hydrophobic interactions are the crucial mechanism behind structure stabilization in thermostable proteins. It has been shown that every additional methyl group inserted into a protein structure on average increases the stability of the folded protein conformation by ~1.3 (±0.5) kcal/mol [31].

**Ion pairs and binding to metal ions**

Comparison of the spatial structures of thermophilic proteins and their analogues from mesophilic organisms has demonstrated that thermophilic proteins have a significantly higher number of ion pairs, which considerably stabilizes their structure at high temperatures [32]. One of the most vivid illustrations of this phenomenon is the content of ion pairs observed in hyperthermophilic lumazine synthase from *Aquifexae olicus,* which was more than 90% higher than that in its mesophilic analogue from *Bacillus subtilis *[33].

It has been known for a long time that metal ions often stabilize and activate certain enzymes. Hence, xylose isomerase from *Streptomyces rubiginosus *binds to two ions from the set of Co^2+^, Mg^2+^ or Mn^2+^: one of them is directly involved in catalysis, while the other one predominantly participates in the stabilization of the enzyme structure [34]. Some thermostable ferredoxins have been shown to contain metal ions that are not found in their mesophilic homologues [35].

**Increase in the number of noncovalent interactions**

It is believed that an increase in the nonlocal noncovalent interactions (e.g., ion pairs, hydrogen bonds, and van der Waals contacts) binding the non-adjacent portions of proteins can significantly increase their thermal stability. Recently accumulated data generally prove this hypothesis. Hence, chimeras are built using the homologous proteins rubredoxins from *Pyrococcus furiosus *and *Clostridium pasteurianum* [36]. The relative stability of these chimeras as compared to rubredoxins from *P. furiosus *and *C. pasteurianum* indicate that there are interactions between the protein nucleus and one of the β-sheets via hydrogen bonding and hydrophobic interactions, which makes a considerable contribution to the thermal stability of the protein. Neither the nucleus nor the β-sheet separately ensures the required stabilization. A comparison of identical proteins from the thermophilic and mesophilic organisms (373 protein pairs http://phys.protres.ru/resources/ termo_meso_base.html) has shown that the former have a closely packed water-accessible residues, while the packing of the interior parts of these proteins are almost identical in both cases [37].

**Hydrogen bonds**

Another factor of the type is the formation of additional hydrogen bonds that stabilize the structure of a number of thermostable proteins at high temperatures [38, 39, 40]. In particular, an investigation of the mechanism of action of hydrogen bonds on the thermal stability of RNAse T1 has shown that every additional hydrogen bond increases the thermal stability of this protein by on average 1.3 kcal/mol [38]. Tanner et al. [39] revealed a strong correlation between the thermal stability of the GAPDH protein (glyceroaldehyde- 3-phosphate dehydrogenase) and the number of hydrogen bonds between the polar uncharged amino acid residues in it. An assumption was made that there are two main reasons that explain what effect the existence of additional hydrogen bonds may have on the thermal stability of the protein: 1) the dehydration energy of these residues is much lower than that of the charged residues in ion pairs, and 2) the gain in enthalpy for these hydrogen bonds is significantly higher due to electrostatic charge–dipole interactions.

**Formation of disulfide bridges**

Formation of additional disulfide bridges is another crucial factor that stabilizes the protein structure at high temperatures [41, 42]. This effect is believed to be for the most part related to the reduction of the configurational entropy of the unfolded protein state.

In some cases, the effect of inserting multiple disulfide bridges into the structure was additive [43]. In particular, mutants with disulfide bridges between the residues 3–97, 9–164 and 21–142 were designed in the bacteriophage T4 lysozyme molecule (the disulfide-free enzyme), which turned out to be significantly more thermostable than the wild-type protein.

However, no such additivity was observed in other cases [42, 44, 45]. Furthermore, formation of disulfide bonds sometimes has no effect on the thermal stability of a protein [45] or even reduces it [42], thus an indication that there are regions with different thermal sensitivities in a protein’s structure. Interestingly, the magnitude of the effect of thermal stabilization of a protein using artificial disulfide bridges, at least in some cases, is approximately proportional to the logarithm of the number of amino acid residues that separate two cysteine residues forming a disulfide bridge [16, 43].

This approach to designing thermostable proteins has recently acquired additional popularity due to the elaboration of novel theoretical approaches that allow one both to calculate all the possible combinations of artificial disulfide bridges based on the known spatial structure of the protein and to roughly estimate their energy and the probability of spontaneous formation [46].

**Directed evolution**

Directed evolution is the main experimental method used to improve enzyme properties [47]. The key advantage of this approach is that it does not require any knowledge about the details of the structure of the enzyme being altered. The method is based on the experimenter- controlled process of artificial, accelerated evolution of microorganisms that are intentionally exposed to harsh environmental conditions. As opposed to natural evolution, this process is more intense and selective; it has a specific purpose, is time-limited, and imitates such natural processes as random mutagenesis, recombination, and selection.

Research into directed evolution of industrially relevant proteins using chemical and radiation-induced mutagenesis was started in the early 1980s. In the 1990s, it accelrated as the era of industrial molecular biotechnology began. The intense development in this field is driven by the demand to produce new natural biocatalysts that would be more efficient and safe for humans. A novel approach, the DNA shuffling method, was proposed in 1994 [48]: it proved to be efficient and underlay a number of different modifications of the original method. Hence, this approach was used to produce thermostable subtilisin E, which is 15 times more active at 37°C than the wild-type protein from *B. subtilis *[49]. The examination of its structure showed that most of the mutations that increase the thermal stability of the protein reside in the loops connecting secondary-structure elements [50]. In a different experiment, the thermal stability and activity of *p*-nitrobenzyl esterase from *B. subtilis *were increased by five low-accuracy PCR cycles accompanied by one DNA shuffling step [51]. The thermal stability of the mutant protein increased by 10°C; its activity was higher than that of the wild-type enzyme at any temperature.

## COMPUTATIONAL METHODS FOR A RATIONAL DESIGNING OF THERMOSTABLE PROTEINS

A number of theoretical models and computer-assisted algorithms based on physical and chemical principles have been proposed to predict the conformational stability of proteins and to design thermostable mutants [52, 53, 54]. The results demonstrate rather convincingly that these approaches may become reliable tools of protein engineering in the near future.

The molecular dynamics method (MD) is one of the well-proven computational approaches to the simulation of the conformational mobility of proteins and their folding into the native conformation, as well as to the rational design of proteins with altered properties [55]. In order to eliminate the need to simulate excessively long molecular dynamics trajectories, a theoretical model and the corresponding software have been developed which allow one to predict the mobile and more stable regions in a protein with a known spatial structure without simulating the detailed molecular dynamics of this protein [56].

Multiple MD trajectories of the same protein under identical conditions make it possible to determine the structure and sequence of its intermediate states during thermal unfolding [57]. These observations can provide hints about how the unfolding of the enzymes starts and which enzyme regions are the most suitable for stabilization [58].

Other approaches based on modern methods for iteration and optimization of the energy of the side-chain conformational isomers of the amino acid residues in proteins under study are also used besides MD [59]: for example, the computer-assisted global optimization algorithm based on the DEE theorem imposing conditions for revealing the rotamers that cannot be members of the conformation characterized by the global energy minimum [60]. This approach was employed to design a thermostable mutant of streptococcal protein G [61]. The melting point of the mutant protein was beyond 100°C, which is 20°C higher than that of the wild-type protein.

The energy potential for assessing the effect of point mutations on the stability of globular proteins with known spatial structures has recently been developed [62]. These computations are also available online (http://foldx.embl.de/). The computations include an assessment of changes in the free energy of the protein after amino acid substitution and the calculated position of charged groups, water bridges, and metal binding sites, which can also greatly affect the conformational stability of proteins.

## FACTORS AFFECTING THE CONFORMATIONAL STABILITY OF α-HELICES IN SHORT PEPTIDES

Unlike in proteins, short peptides 10- to 20 amino-acid- residues-long lack many of the possibilities for structure stabilization that globular proteins have. Back in the early 1980s, it was thought that short peptides cannot maintain their stable conformation in water even at relatively low temperatures [63]. However, in the mid-1970s, Finkelstein and Ptitsyn predicted in their theoretical studies that short peptides consisting of amino acids exhibiting high proneness to the α-helical structure can have appreciably stable α-helical conformations in aqueous solutions [64, 65, 66, 67, 68]. These theoretical predictions were later experimentally proven by investigating synthetic peptides whose sequences repeat those of ribonuclease A α-helices [69, 70]. The theoretical model developed by Finkelstein and Ptitsyn describes the probabilities of formation of α-helices, β-structures, and turn regions in short peptides and globular proteins based on the classical Zimm-Bragg approach, with modifications that take into account some additional interactions, such as the electrostatic interactions between the charged side chains and the macrodipole of the α-helix, as well as the hydrophobic interactions between the side chains of certain amino acids. This theoretical model was employed to design software (ALB) that successfully predicts both the approximate level of conformational stability of α-helical peptides [4] and, with a ~65% probability, the distribution of the secondary structural elements in globular proteins [71]. The main drawback of this model is that it does not take into account certain interactions (e.g., the so-called Capping Box and/or presence of proline in the first N-terminal position of α-helix), the positional dependences of the propensities of amino acids in the first and last turn of an helix, or the effect of various special sequences of the regions in the peptide under study that are adjacent to the α-helix (as demonstrated later, these regions play a significant role in the stabilization of the α-helical conformation in proteins and peptides).

Starting in the late 1980s, and especially in the 1990s, a large number of experiments have been conducted where amino acid substitutions in short synthetic polyalanine- based peptides were used to study various interactions in α-helices [72]. This approach has allowed researchers to accumulate sufficient data and to proceed to a quantitative description of the cooperative mechanisms of conformational transitions into the α-helical conformation for peptides with random sequences [5, 73].

It is currently believed that each of the 20 natural amino acids has an intrinsic propensity to form α-helical conformations in peptides and proteins that is associated with their covalent structure (e.g., changes in the configurational entropy of the side chains of amino acids during a transition from a random conformation into the α-helical one) [74]. In addition, the stability of α-helical protein conformations is affected by the interactions between side chains (between the residues at positions *i,i+*3 and *i,i+*4), the electrostatic interactions between the charged polar residues with the macrodipole of the α-helix, and the capping interactions between the residues adjacent to the α-helix and the unbound NH- and CO- moieties in the main chain of the protein in the first and last turn of the α-helix [5, 73].

Furthermore, local motifs of amino acid sequences that include the residues adjacent to the α-helix, which either are specifically packed against the residues of the first (N-terminal) and last (C-terminal) turn of the helix or form a network of specific hydrogen bonds with it, have also been reported [75].

**Fig. 1:**
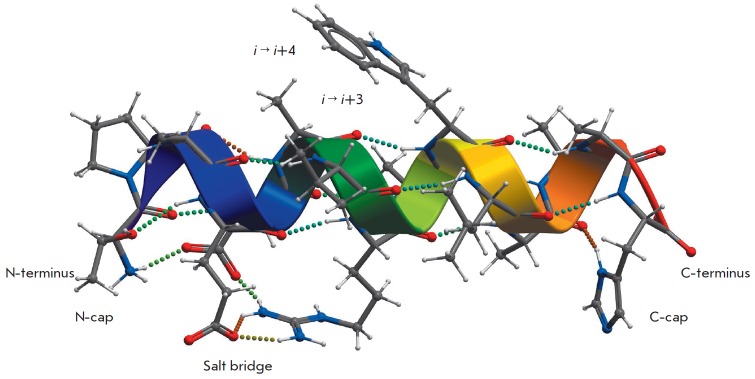
The structure and factors that influence the conformational stability of the α-helix in proteins and monomeric peptides

It is usually assumed that the structural propensity of amino acids is independent of their positions in the α-helix [4, 76, 77]. However, the first and last turns of the α-helix are not equivalent to the remaining portion of the helix. *Figure 1*shows that the mobility of the side chain of valine at the central positions of the helix is strongly limited compared to the situation when it resides are in the first turn of the helix. The accuracy of the theoretical models of α-helix/random coil transitions in the description of experimental findings on measuring the stability of α-helical peptides with complex amino acid sequences is significantly reduced if no allowance is made for this factor [78, 79, 80].

## CHEMICAL METHODS FOR THE STABILIZATION OF α-HELICAL STRUCTURES

Since α-helices often serve as a structural basis for intermolecular interfaces of protein complexes, they are frequently used to design peptide inhibitors targeted against the formation of these complexes. Targeted destruction of certain protein–protein interactions using α-helical or β-structural peptides is a topical issue in chemical biology that researchers are currently trying to solve.

**Fig. 2:**
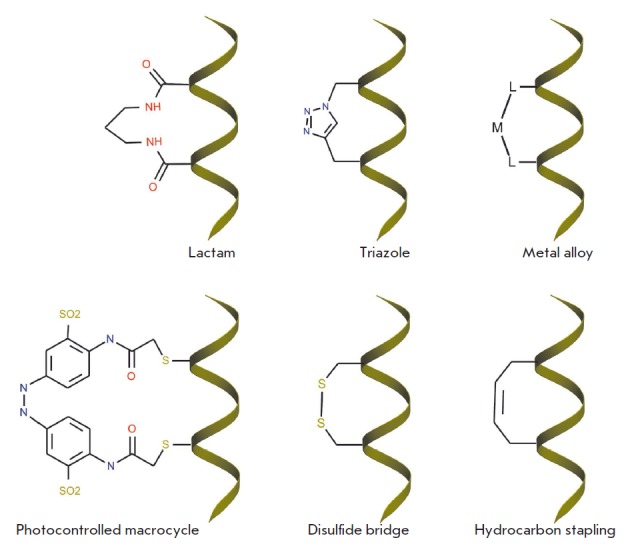
The main ways of chemical modification to increase the conformational stability of α-helical structures

However, the use of peptide inhibitors has serious limitations. First of all, there is the insufficient stability of the α-helical conformation of short peptides with amino acid sequences isolated from natural proteins. Furthermore, these proteins are characterized by poor cell membrane penetrability and are easily degradable by proteases. Over the past 30 years, various methods for chemical modification of short α-helical peptides have been designed to increase the stability of α-helical conformations and their proteolytic stability (*Fig. 2*). Chemical modifications for stabilizing α-helical conformations include: 1) inserting residues with limited mobility, such as α-α-dialkylated residues [81], into the amino acid sequence; (2) covalent crosslinking of side chains of the amino acids residing on neighboring turns of α-helices, including the formation of covalent bridges based on disulfide bonds [82], lactam structures [83] and hydrocarbons [3]; and 3) capping the group at the N- or C-termini of the peptide [84], as well as various combinations of the aforementioned modifications [2].

The chemical modifications that stabilize α-helices turn out to also be able to improve the cell permeability of these peptides in some cases, making them good inhibitors of an intracellular target. In particular, a large body of data on increased membrane permeability in some types of human cancer cells for chemically modified α-helical peptides has been published [85].

## DESIGNING STABLE α-HELICES OF PROTEINS THROUGH GLOBAL OPTIMIZATION OF THEIR AMINO ACID SEQUENCES

SEQOPT, the recently developed method for a global optimization of the amino acid sequences of α-helices in monomeric peptides and globular peptides is one of the efficient methods for solving the problem associated with the stabilization of the structure of biologically active α-helical peptides using natural amino acids only [1]. This method allows one to design α-helices of proteins that have the maximum possible conformational stability under specific conditions (conformational environment, pH, temperature, and ionic strength of solution) using global optimization of amino acid sequences, including arbitrary fixation of any amino acid combinations. The model that theoretically underlies the proposed method is the AGADIR model, which describes the thermodynamics of folding of α-helices under various ambient conditions (temperature, pH, and ionic strength of solution, etc.) [77] and has also been used to design mutant proteins that exhibit enhanced conformational stability [7]. Its model reproduces well the existing experimental data on the stability of the α-helical conformations of a large number of short peptides [73, 77, 78, 79, 80, 86, 87, 88].

The dependence of the energy parameters of the model on the temperature, pH, and ionic strength of the solution was included in the calculations as described in [86].

Although no guaranteed convergence to the global minimum can currently be achieved for the majority of multivariate problems that are of practical significance, the elaborated method was shown to be characterized by high efficiency in optimizing the amino acid sequences of α-helical peptides. The measured CD values of several synthetic peptides with optimized sequences demonstrated good agreement with theoretical calculations in terms of both the absolute and relative α-helical contents [6].

**Fig. 3:**
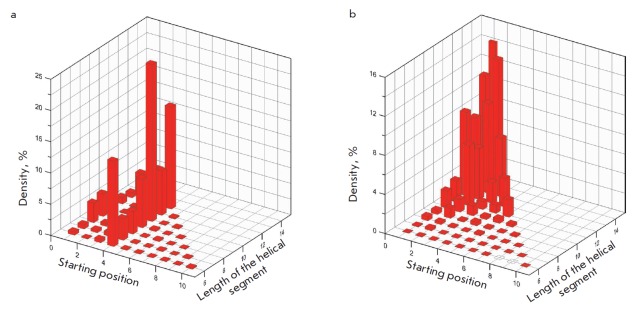
The distribution of populations of all possible segments in a short peptide 13-amino-acid-residues-long (according to AGADIR [77]). *A *– the C-terminal peptide from ribonuclease A (ac-AETAAAKFLRAHA-nh2) [69, 70];* B *– the peptide with an optimized sequence of the same length ac-DYMERWYRYYNEF-nh2

It is well-known that short peptides are typically rather mobile and do not have a single dominant conformation. *Figure 3* shows the distributions of populations for all possible segments in 13 amino acid residues short peptides. The sequence (AETAAAKFLRAHA) of one of these peptides (see panel *3A*) corresponds to the α-helix of ribonuclease A, one of the first peptides whose significant stability of the α-helical conformation in water has been demonstrated experimentally (HC ~21%, 5°C, pH 7, ionic strength 100 mmol/L, the N- and C-termini are acetylated and amidated, respectively). The data for a peptide of the same length but with the optimized sequence DYMERWYRYYNEF and HC ~ 88% are shown in *Fig. 3B* for the sake of comparison.

These figures demonstrate that in the protein with the amino acid sequence taken from the globular protein, several helical segments starting with alanine at position 4 were populated in the solution. The populations of each segment changed randomly depending on its length and, therefore, the amino acids of the C-terminal portion of this region. As a result, the first four and the last two amino acids in this peptide stand almost no chance of participating in the formation of α-helical conformation, whose average length is ~6 amino acid residues. Therefore, the helical content of this peptide is rather low: about 21%.

The optimized sequence, as opposed to the native one, behaves in completely different fashion. The helical segment covering the entire peptide sequence is characterized by the highest population. It is followed by segments differing from the maximum segment by one or two residues that have lost their α-helical conformation at the N- and C-termini.

As a result, the total helical content of the peptide with the optimized sequence is as high as ~90%. The stability of the α-helical conformation rises with increasing peptide length and approaches 100%. The average length of the α-helical region of the peptide is also considerably greater. These results both demonstrate the potential of the SEQOPT method and indicate that the potential of 20 natural amino acids allows one to obtain appreciably stable conformations in the short α-helical peptide that are as short as 10–20 residues.

**Fig. 4:**
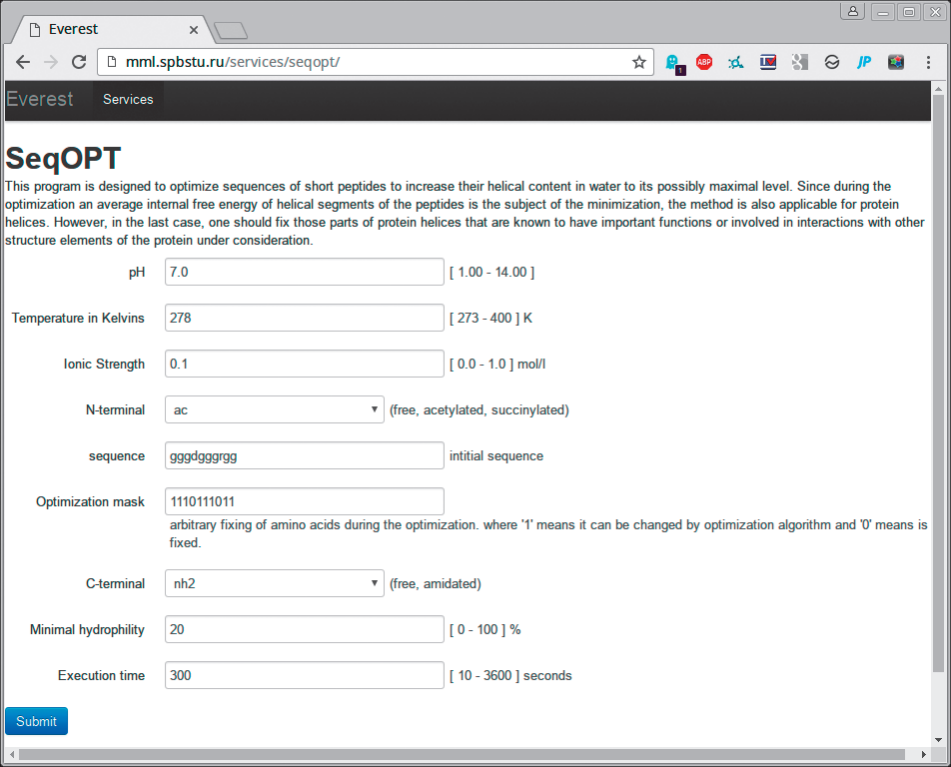
The screenshot of the SEQOPT software for specifying parameters, such as pH, temperature, ionic strength, the initial sequence for the optimization and the fixed position of amino acid residues, the minimum level of solubility, and the allowed calculation time

A new function of the algorithm that is of practical importance has been added in the updated SEQOPT version (web server can be accessed at http://mml.spbstu.ru/services/seqopt/, see *Fig. 4*). It enables to determine the minimal allowable level of solubility for α-helical peptides with optimized sequences. As far as the authors know, it is the first study in the new and promising field of global optimization of the amino acid sequences of proteins.

## PERMEABILITY OF CELL MEMBRANES TO PEPTIDES

The highly stable α-helical peptides that are employed as highly active and specific inhibitors of protein–protein interactions are currently being used with increasing regularity in medicine as broad-spectrum antibiotics and to destroy certain complexes that play a key role in the activity of human cells [2]. One of the key downsides in using peptides in medicine consists in their penetrability through the cell membranes.

The cell wall prevents the penetration of foreign molecules inside the cell, thus impeding the use of the designed highly stable peptides for therapeutic purposes. There are several approaches to solving this problem. One of these approaches is based on using special receptors that recognize certain chemical compounds and switch on the mechanisms of active transport inside the cell [89]. Another method consists in the destruction of the cell membrane and penetration through the newly opened pores.

**Table 1:** The most commonly used peptides that exhibit antibacterial activity and can penetrate through the cell membrane

PEPTIDE	AMINO ACID SEQUENCE	SECONDARY STRUCTURE	REFERENCE
Penetratin	RQIKIWFQNRRMKWKK	α-helical	[90]
Tat	GRKKRRQRRRPPQ	nonstructural, PPII-helical	[91]
Pep-1	KETWWETWWTEWSQPKKKRKV	α-helical	[92]
S4_13_-PV	ALWKTLLKKVLKAPKKKRKV	α-helical	[93]
Magainin 2	GIGKFLHSAKKFGKAFVGEIMNS	α-helical	[94]
Buforin II	TRSSRAGLQFPVGRVHRLLRK	α-helical	[95]
Apidaecins	RP - - - - - PRPPHPR	nonstructural	[96]
Transportan (TP10)	GWTLNSAGYLLGKINLKALAALAKKIL	α-helical	[97]
MAP	KLALKLALKALKAALKLA	α-helical	[98]
sC18	GLRKRLRKFRNKIKEK	α-helical	[99]
LL-37	LLGDFFRKSKEKIGKEFKRIVQRIKDFLRNLVPRTES	α-helical	[100]
Bac-7	PFPRPGPRPIPRPLPFPRPGPRPIPRP	PPII- and α-helical	[101]

The entire family of peptides that exhibit antimicrobial properties, can penetrate through cell membranes, and are able to carry both other peptides and chemical compounds of a different nature through the membrane is known and well-studied [102, 103]. These peptides were isolated from proteins of various organisms, ranging from viruses to higher organisms (*Table 1*).

**Table 2:** N-terminal peptides facilitating the penetration of microorganisms into cells

AMINO ACID SEQUENCE	Candida albicans	Saccharomyces cerevisae	Staphylococcus aureus	Bacillus subtilis	Escherichia coli	Reference
VLTNENPFSDP	+		+	+		[106]
YKKSNNPFSD		+		+	+	[107]
RSNNPFRAR	+	+	+			[107]
CMVSCAMPNPF					+	[108]
LLDLMD	+					[109]
LMDLAD	+				+	[109]
RQIKIWFQNRRMKWKK	+					[110]
YGRKKRRQRRRCKGGAKL			+			[110]
CFFKDEL					+	[111]
GASDYQRLGC		+			+	[111]

Successful use of peptides that exhibit antibacterial activity to deliver therapeutic agents inside the cell has been demonstrated in a number of experiments [101, 104, 105]; there is no fundamental difference in the efficiencies of their penetration into different cells. Signal peptides belonging to another group can also penetrate into these cells. The common mechanism of their action is still to be determined [89]. *Table 2* lists the amino acid sequences of peptides and indicates their ability to penetrate into the cells of single-celled microorganisms.

These peptides can be synthesized or cloned along with the required therapeutic agents.

## INTRACELLULAR PROTEOLYSIS OF Α-HELICAL PEPTIDES

One of the key issues hindering the development of peptide therapeutic agents is their proteolytic instability and the problems associated with delivery to molecular targets. Proteolysis typically takes place in the intestine, in microvilli on the inside walls of the small intestine, in enterocytes, hepatocytes, antigen-presenting cells, and plasma; hence, oral administration of peptide-based drugs is usually infeasible and injections are needed. However, even in the case of parenteral administration, the degradation of peptide-based drugs in the blood, in combination with rapid renal clearance, makes this type of therapeutic agents expensive and inconvenient [112]. Furthermore, synthetic therapeutic peptides are typically non-structured and, therefore, are cleaved rapidly by intracellular proteases under natural conditions; their half-life often does not exceed a few minutes.

The proteolytic stability of α-helical peptides can be increased by inserting various factors that stabilize the conformational stability of the α-helix: additional saltbridge bonds or other modifications, such as lactam bridges [113, 114], or formation of peptide oligomeric structures [115].

Since natural peptides are in general characterized by a relatively short lifetime in plasma, several approaches have been designed to increase it. The first approach is aimed at limiting enzyme degradation by identifying the possible peptide cleavage site, followed by structural modifications, such as amino acid substitution at the cleavage site. Peptide resistance to cleavage can also be enhanced by improving the peptide’s secondary structure folding. This approach involves the use of structure-induced probes: the (SIP)-tail s, lactam bridges, and either stapling or cyclization of the peptide chain [3, 83, 116].

Another method used to increase a peptide’s lifetime is to bind the peptide to circulating protein albumin as a transporter and peptide acylation [117]. Binding of polyethylene glycol to peptides is often used to increase plasma elimination the half-life of peptide-based agents [118].

## CONCLUSIONS

Biologically active peptides are becoming increasingly popular as potential therapeutic agents because of their high activity, nontoxicity, and moderate cost. The problems related to their insufficient conformational stability, penetrability through cell membranes, and rapid degradation by intracellular proteases are overcome to a significant extent through employing modern methods for the design of highly stable peptides based only on natural amino acids or using several types of their chemical modifications. SEQOPT is a recently developed computational method for designing α-helical peptides that contain only 20 natural amino acids. Peptides with the maximum possible stability of α-helical conformation can be produced using this method. It allows one to take into account the conformational environment, the ambient conditions (pH, temperature, and ionic strength of solution), and the minimum acceptable solubility level and to arbitrarily fix any amino acid combinations needed to ensure the biological activity of the peptides. The conformational stability of monomeric peptides with an optimized structure approaches that of the α-helical regions of the secondary structure of globular proteins.

## Abbreviations

SEQOPT: method for α-helix amino acid sequence optimization
MD: molecular dynamics method
HC: α-helix content
PDB: protein database
AGADIR, ALB, HELIX: the statistical mechanical models describing the conformational α-helix–coil transitions in short monomeric peptides
CD: circular dichroism spectroscopy
